# Does psychological status influence clinical outcomes in patients with inflammatory bowel disease (IBD) and other chronic gastroenterological diseases: An observational cohort prospective study

**DOI:** 10.1186/1751-0759-2-11

**Published:** 2008-06-06

**Authors:** Antonina A Mikocka-Walus, Deborah A Turnbull, Nicole T Moulding, Ian G Wilson, Gerald J Holtmann, Jane M Andrews

**Affiliations:** 1 Discipline of General Practice, University of Adelaide, Level 3, Eleanor Harrald Building, Adelaide 5005, SA, Australia; 2 School of Psychology, University of Adelaide, Level 4, Hughes Building, Adelaide 5005, SA, Australia; 3 School of Social Work and Social Policy, University of South Australia, Magill Campus, H1-32, Magill 5068, SA, Australia; 4 School of Medicine, University of Western Sydney, Locked Bag 1797, Penrith South DC NSW 1797, Australia; 5 Department of Gastroenterology and Hepatology, Royal Adelaide Hospital, North Wing Q7, Adelaide 5005, SA, Australia; 6 Department of Epidemiology & Preventive Medicine, Monash University, The Alfred, Level 3, Burnet Tower, 89 Commercial Rd, Melbourne 3004, VIC, Australia

## Abstract

**Background:**

Whether there is a temporal relationship between psychological problems and clinical outcomes in patients with diseases of the digestive tract has not been widely researched. Thus, our aims were 1) To observe and compare prospectively clinical outcomes in relation to psychological co-morbidity in patients with inflammatory bowel disease (IBD), irritable bowel syndrome (IBS) and chronic hepatitis C (HCV) and, 2) To test the hypothesis that patients with psychological co-morbidities are less likely to have a satisfactory response to standard treatment at 12 months.

**Methods:**

Overall, 139 patients were enrolled in this observational cohort prospective study. Over the ensuing year, physical and psychological measures were made at baseline and after 12 months (HADS, SCL90, SF-12 and disease activity measures). A logistic regression was conducted to observe any relationship between baseline characteristics and patients' clinical outcomes after 12 months.

**Results:**

Overall, there was no relationship between psychological status and quality of life at baseline and relapse at 12 months (p > 0.05). However, patients with inactive disease at baseline were at lower risk of relapse after 12 months (OR = 0.046, CI: 0.012–0.178). No significant relationship was found between psychological problems such as depression/anxiety and a total number of relapses in the IBD group. However, interestingly, patients with an active disease at baseline tended to have a greater number of relapses (OR = 3.07, CI: 1.650–5.738) and CD participants were found at lower risk of relapse than UC participants (OR = 0.382, CI: 0.198–0.736).

**Conclusion:**

In contrast to previous investigations, this study suggests that there is no temporal relationship between psychological problems at baseline and clinical outcomes over time. Longer and larger prospective studies are needed to better understand this result.

## Background

Diseases of the digestive tract frequently coexist with psychological disorders [1-3]. However, a temporal relationship between psychological problems and clinical outcomes in patients with gastroenterological disorders has not been widely researched.

Inflammatory bowel disease (IBD) is a generic term used to describe a group of chronic relapsing inflammatory disorders of the gastrointestinal tract, of which Crohn's disease (CD) and ulcerative colitis (UC) are the most common. The prevalence of IBD ranges from 37 to 246 cases per 100,000 persons for UC and from 26 to 199 cases per 100,000 persons for CD depending on the region of the world [4], with a peak incidence around 20 years of age. The aetiology of IBD is unknown. Nonetheless, genetic, immune and environmental factors have all been implicated in its causation [5,6]. Some researchers have also controversially proposed that IBD may be partly a psychosomatic disease [7-11]. However, the editors of major texts in gastroenterology claim that psychological factors are a result, rather than a cause of IBD, and that they do not contribute to the aetiology [12,13]. Although the possible psychosomatic origin of IBD is disputed, many studies report that stressful life events do exacerbate the disease [14-17].

Consistent with these observations in IBD, in the majority of other gastrointestinal problems, depression and anxiety are considered to be a consequence of chronic somatic disease or treatment side-effects (e.g. interferon-ribavirin and depression) rather than causal factors [18]. The exception may be functional gastrointestinal disorders which some authors believe have psychological factors implied in their causation [19]. However, even in this group of conditions there has been little prospective research conducted on the relationship between psychological status at baseline and clinical outcomes over time.

In patients with irritable bowel syndrome (IBS), which is the most well recognised of the functional gastrointestinal disorders, bowel habits are altered (constipation and/or diarrhoea) with absence of any apparent mechanical, biochemical and inflammatory changes in the gastrointestinal tract [20]. Surprisingly, a prospective study of 400 IBS participants has found that anxiety, depression and stress are all predictors of better health outcomes over a 12 month period [21]. Whereas another 5-year follow-up study with 43 participants has indicated that anxiety but not depression may have a negative impact on the clinical outcomes in IBS [22]. In contrast, a systematic review including 14 observational longitudinal studies has documented that both anxiety (two studies including the one by Fowlie et al. 1992) and depression (one study) at baseline predicted worse clinical outcomes after a period of observation [23]. These somewhat conflicting results make it impossible to confidently conclude whether psychological status affects clinical outcomes in patients with IBS and whether the effects are positive or negative.

Prospective studies on the relationship between psychological status and clinical outcomes in chronic hepatitis C (HCV), a chronic infectious disorder that carries a mortality risk from cirrhosis, end-stage liver disease and liver cancer [24], and IBD are even more of a rarity. In fact, searching for prospective studies on depression in HCV, among the 49 results, the only papers found all relate to depression as a side-effect of interferon therapy. With regard to IBD, among 18 identified studies, the majority relate to stress and psychotherapy, with only three prospective investigations into the relationship between depression/anxiety and clinical outcomes [25-27]. The two largest of these three studies have demonstrated a link between psychological problems and poorer clinical outcomes [26,27], whereas a study with a small sample (n = 32) but with the longest follow-up period did not confirm this finding [25].

Moreover, the majority of controlled studies in this area suffer from methodological flaws such as comparisons with inadequately matched controls. In fact, among the three available studies on the relationship between depression and clinical outcomes in IBD, none is controlled [25-27]. The present study aims to avoid this limitation.

The innovative aspects of the present study are therefore: the groups under investigation; its prospective design, comparisons with appropriate disease controls and; most importantly, its focus on the relation between anxiety, depression and clinical outcomes. Furthermore, as the temporal differences in psychological profiles, the quality of life and disease activity between patients with inflammatory bowel disease, irritable bowel syndrome, and hepatitis C have not been previously examined, discovering their character and directions might contribute to understanding the nature of problems affecting these patients and consequently, to improved medical care. The following hypothesis is investigated in this study:

- Patients with psychological co-morbidities are less likely to have better clinical outcomes (remission) at 12 months.

## Materials and methods

### Participants and recruitment

Patients with clinically diagnosed IBD, IBS and HCV were recruited to this observational cohort prospective study between November 2005 and June 2006 through the Outpatient Clinic at the Department of Gastroenterology and Hepatology at the Royal Adelaide Hospital. Participants each had sufficient knowledge of English to understand and answer the questionnaires. Gastroenterology clinic consultants individually invited eligible patients to participate. After obtaining patients' consent, consultants passed patients' contact details to the first author who then made contact and provided written information regarding the study. The study was described to potential participants and each participant was provided with a consent form, questionnaires and a reply paid envelope. Through the year of the study, participants were administered a number of screening instruments: IBS and HCV patients on two occasions (baseline and 12 months) and IBD patients on five occasions (baseline, 3, 6, 9 and 12 months). At each of these time points, participants were asked to complete a survey comprising a measure of anxiety and depression; a measure of a broad psychological profile; a measure of quality of life; and a measure of disease activity (or in the case of patients with HCV, a blood test to assess for ongoing active viral replication). Participants were given a choice to complete the questionnaires at hospital or at home.

### Measurements

#### Anxiety and depression screening

Screening for anxiety and depression was conducted with the Hospital Anxiety and Depression Scale (HADS). The HADS contains 14 questions graded on a 4-point Likert scale (0–3), with subscales of anxiety (seven items) and depression (seven items), with a sum score ranging from 0 to 21. A cut-off value for clinical caseness is 7. Scores between 8 and 10 are interpreted as possible cases, and ≥ 11 as certain cases.

#### Psychological profiles

Participants' broad psychological profiles were assessed by the SCL-90-R Symptom Checklist (SCL-90). This is a 90-item self-report instrument. The SCL-90 contains 9 subscales: Somatization, Obsessive-Compulsive, Depression, Anxiety, Hostility, Phobic Anxiety, Paranoid Ideation, and Psychoticism. It also comprises three global indices: Global Severity Index (GSI), Positive Symptom Distress Index, and Positive Symptom Total [28]. There are no specified cut-off values for this scale; however, caseness can be identified when the GSI score ≥ 63 (after the transformation into the T score) or when any two primary dimension scores are ≥ 63.

#### Quality of life

Screening for quality of life was performed with the use of the Short Form 12 Health Survey (SF-12). The SF-12 contains two subscales: the Mental Component Summary (MCS) and the Physical Component Summary (PCS) [29]. Scores for each subscale range between 0 and 100, with increasing values indicating better health.

#### Disease activity and the definition of remission

Disease activity in IBD participants was assessed with the Crohn's Disease Activity Index (CDAI) [30] or the Simple Clinical Colitis Activity Index (SCCAI) [31] as appropriate. A CDAI score ≤ 150 was considered remission in CD and a SCCAI score of ≤ 2 was considered remission in UC; thus CDAI scores of more than 150, and SCCAI scores > 2 were considered a relapse (active disease).

Disease activity in IBS patients was measured by two questions added into the general health survey: "Have you got satisfactory control of your IBS symptoms over the last 3 months?" and "Are you now feeling better or worse when compared to your last visit in the clinic?" Answering "yes" and "better" to these questions was considered remission in IBS. Other responses were considered a relapse (active disease). These questions were used as IBS is a symptom-based diagnosis, according to a patient report (the Rome criteria). The three month time frame was selected as it corresponds to published diagnostic measures [32]. Given the symptom based expression of this disease, "satisfactory control" as judged by the patient is arguably the most valid single tool to assess relapse/remission status.

Disease activity in HCV (or, more precisely, active viral replication) was measured by RT-PCR HCVRNA. RT-PCR HCVRNA "Not detected" was considered remission of HCV and "Detected" was considered a relapse (active disease).

The primary outcome measure in this study was remission (inactive disease) as compared to relapse (active disease).

#### Standard medical treatment

Standard medical treatment included regular visits at the clinic and the medication prescribed by the treating doctor. No interventions were made. Further appointments were not "regular" they were at discretion of the referring doctor according to medical needs.

### Ethical considerations

The study was approved by the Royal Adelaide Hospital Research Ethics Committee. Participants were aware that their care did not in any way depend on participation or non-participation in the study. Each participant provided written informed consent. The work was performed in accordance with the principles of the 1983 Declaration of Helsinki [33].

### Statistical analysis

Powering the study to detect differences of more than 15% for the primary outcome variables with an alpha-level of 0.05 and a beta-level of 0.86 a sample size of at least 150 patients (50 in each patient group) was estimated to be acceptable for this trial. Estimates were based on the published rates of psychological co-morbidities [26,27,34,35]. However, due to the lack of good quality data in this area and absence of local data, this power calculation was an educated guess and was regarded as an indication only. In terms of recruitment, the expected sample sizes were larger and it was planned to recruit 50 to 125 participants to the IBD group and 50 to 75 participants to each of the IBS and HCV groups. The intended total sample (for IBD, IBS and HCV) was 150 – 275 patients.

A logistic regression with a binomial dependent variable was conducted to observe a relationship between baseline characteristics (Point 0) and patients' clinical outcomes after 12 months (Point 4). The analysis was adjusted for disease activity at baseline, sex, years since diagnosis and age. In order to obtain more parsimonious results, after building a general model for all the psychological variables versus relapse, data were explored as part of a model with significant and demographics variables only. A Poisson regression analysis was conducted to observe whether there was any relationship between baseline characteristics and the total number of time points at which the IBD group had active disease (were in relapse). Demographic comparisons and comparisons between CD and UC participants were included into the analysis.

## Results

Overall, 139 patients were enrolled in this observational cohort prospective study. After 12 months of the study, 13 (9.3%) participants withdrew. These included seven patients with IBD, five patients with HCV and one patient with IBS. Five participants were not contactable, three were too sick to be able to participate (Cancer, Alzheimer disease, serious relapse of IBD). Another three participants did not send back the questionnaires despite the reminders. One IBS patient's diagnosis was changed to Coeliac disease and one person did not wish to be involved anymore. Thus, 126 (90.7%) participants completed the study. Only incomplete data were available for two IBS patients. The analysis was, therefore, restricted to 124 participants (59 IBD patients [32 CD and 27 UC], 36 HCV and 29 IBS). Unfortunately, the study was underpowered for the HCV and IBS group, as the estimated sample size of at least 50 patients in each disease group was not achieved in these two groups.

Overall, 33 IBD participants received treatment with immunosuppressant and eight patients received prednisolone. Five HCV patients received antiviral treatment (Interferon-Ribavirin). Overall, 17 patients received antidepressants (6 IBD, 6 HCV and 5 IBS). Moreover, in the HCV group, 10 people did not receive any medication. In the IBS group, three people did not receive any medication and in the IBD group, two people did not receive any medication.

### Baseline characteristics and patients' clinical outcomes after 12 months

Overall, there were 77 (62%) female and 47 (38%) male participants. Groups did not differ in the distribution of sex. The mean age was 50 years (SD = 13), with no difference between groups. The duration of disease was 12 years (SD = 9), with IBD participants having significantly longer disease duration than HCV participants (15 (SD = 10) vs. 8 (SD = 5) years, p = 0.002). Overall, 55 participants (44%) completed secondary education and 38 (30%) completed tertiary education.

Descriptive statistics for the relapse/remission status in all three groups at baseline and after 12 months are presented in Table 1. Both, at baseline and after 12 months, 44% (CI: 35.3–52.7) of participants were in relapse. The median baseline CDAI score was 34 with a range of 0–319. The median baseline SCCAI score was 2.5 with a range of 0–10. There was no difference in the number of participants with active disease at baseline and after 12 months in any of the groups (Table 1).

**Table 1:** The distribution of active disease in HCV, IBD and IBS at baseline and after 12 months

	**Baseline**	**At 12 months**
	Percentage (CI)	
**HCV (n = 36)**	53(36.7–69.3)	36(20.3–51.7)
**IBD (n = 59)**	32(20.1–43.9)	39(26.6–51.4)
**IBS (n = 29)**	59(41.1–76.9)	65(47.6–82.4)
**Total (n = 124)**	44(35.3–52.7)	44(35.3–52.7)

At baseline, 40% participants were anxious and 17% depressed (see Tables 2 and 3). At 12 months, 37% of participants were anxious and 13% depressed. There was no significant change over time either in the anxiety caseness (χ^2 ^(1) = 1.05 p = 0.305) or in the depression caseness (χ^2 ^(1) = 1.11 p = 0.291) for the total cohort. There was also no group differences in either anxiety or depression caseness over time (See Tables 2 and 3). There were no significant differences between groups in the risk of relapse after 12 months as evidenced by the results of logistic regression (Table 4).

**Table 2:** Anxiety cases (HADS Anxiety > 7) at baseline and after 12 months

	**Baseline**	**At 12 months**
	Percentage (CI)	
**HCV (n = 36)**	44(27.8–60.2)	33(17.6–48.4)
**IBD (n = 59)**	39(26.6–51.4)	34(21.9–46.1)
**IBS (n = 29)**	52(33.8–70.2)	48(29.8–66.2)
**Total (n = 124)**	40(31.4–48.6)	37(28.5–45.5)

**Table 3:** Depression cases (HADS Depression > 7) at baseline and after 12 months

	**Baseline**	**At 12 months**
	Percentage (CI)	
**HCV (n = 36)**	33(17.6–48.4)	11(0.8–21.2)
**IBD (n = 59)**	12(3.7–20.3)	15(5.9–24.1)
**IBS (n = 29)**	10(0-20.9)	10(0-20.9)
**Total (n = 124)**	17(10.4–23.6)	13(7.1–18.9)

**Table 4:** Interactions between all psychological, demographic and disease activity variables and the risk of relapse at 12 months in IBD, IBS and HCV patients

	**Adjusted OR**^1^	**95% CI**	**p**
**Disease status at baseline**	0.046	0.012–0.178	<0.001
**Female vs. male**	0.537	0.142–2.033	0.360
**Age**	1.072	1.018–1.129	0.009
**Years since Diagnosis**	0.991	0.931–1.055	0.769
**HADS Anxiety**	1.216	0.929–1.591	0.154
**HADS Depression**	1.110	0.869–1.419	0.403
**SF12 Mental**	1.009	0.936–1.088	0.809
**SF12 Physical**	1.015	0.951–1.083	0.653
**SCL90 Somatisation**	1.045	0.956–1.142	0.330
**SCL90 Obsessive-compulsive**	0.950	0.861–1.048	0.305
**SCL90 Interpersonal sensitivity**	1.070	0.965–1.185	0.199
**SCL90 Depression**	1.014	0.882–1.166	0.843
**SCL90 Anxiety**	0.979	0.874–1.097	0.718
**SCL90 Hostility**	1.009	0.930–1.095	0.825
**SCL90 Phobic anxiety**	1.047	0.961–1.140	0.292
**SCL90 Paranoid ideation**	0.906	0.834–0.985	0.020
**SCL90 Psychoticism**	1.080	0.973–1.198	0.147
**SCL90 GSI**	1.025	0.818–1.283	0.832
**SCL90 PST**	0.936	0.731–1.199	0.602
**SCL90 PSDI**	0.887	0.782–1.005	0.060
**IBS vs. HCV**	1.576	0.371–6.705	0.538
**IBD vs. HCV**	4.940	0.881–27.689	0.069

^1 ^The analysis was adjusted for disease activity at baseline, sex, years since diagnosis and age

As the model did not provide a direct comparison between IBD and IBS patients in their risk of being in relapse after 12 months, an additional contrast was conducted. The groups were found not to differ with this respect (OR = 0.319, CI: 0.072–1.424, p = 0.134).

In the whole cohort, patients with lower scores on the SCL90 Paranoid Ideation subscale were at lower risk of relapse after 12 months than those with higher scores (OR = 0.906, CI: 0.834–0.985 in Table 4). However, no other psychological variable showed any significant relationship to the risk of being in relapse after 12 months. Interestingly, those patients with inactive disease at baseline were at significantly lower risk of being in relapse after 12 months (OR = 0.046, CI: 0.012–0.178). Moreover, older participants were at greater risk of relapse after 12 months than younger participants (OR = 1.072, CI: 1.018–1.129).

When the analysis was rerun with significant and demographic variables only, those patients with an inactive disease at baseline remained at lower risk of active disease after 12 months (OR = 0.089, CI: 0.035–0.229), whilst the significance for the SCL90 Paranoid Ideation subscale disappeared (OR = 0.964, CI: 0.923–1.007, p = 0.099). Again, the p value of 0.025 indicated that older participants were at greater risk of relapse after 12 months; however, the confidence intervals show this difference is barely significant (OR = 1.043, CI: 1.005–1.082).

As the main model comprised a large number of variables potentially overlapping (with many of them being psychological in nature), we subsequently created three smaller models to ensure we were not "over fitting" the data. In each of these three models, we controlled for age, sex, years since diagnosis and disease activity at baseline. In the first model, we examined anxiety, depression (HADS) and quality of life (SF-12). In the second model, we examined all the SCL90 variables. In the third model, we examined only whether a participant met caseness (score > 7) for either anxiety or depression. In all the three models, the variable showing a significant difference was disease activity at baseline. Patients with an inactive disease at baseline were at lower risk of active disease after 12 months (OR = 0.104, CI: 0.040–0.272). There was no relationship between most other variables and relapse at 12 months. However, patients with lower scores on the SCL90 Paranoid Ideation subscale were at lower risk of relapse after 12 months than those with higher scores (OR = 0.900, CI: 0.831–0.974). These findings confirm the negative observations from the above large model and lend support to the conclusion that there is, in fact, no relationship between psychological variables and clinical outcomes in this group of patients.

### Interactions between psychological variables and a total number of relapses in IBD

A total number of relapses in IBD patients at five time points of the 12-month study period are presented in Table 5. No significant relationship was found between psychological problems such as depression/anxiety and the total number of relapses in the IBD group (Table 6). However, interestingly, CD participants (n = 32) were found to have a lower risk of relapse than UC participants (n = 27) (OR = 0.383, CI: 0.198–0.736). Moreover, patients with an active disease at baseline tended to have a greater number of relapses (p < 0.0001) (OR = 3.07, CI: 1.650–5.738). We subsequently created two smaller models to confirm these findings. We controlled for age, sex, years since diagnosis, IBD subtype and disease activity at baseline. In the first model, we examined anxiety, depression (HADS) and quality of life (SF-12). In the second model, we explored SCL90 subscales. The only significant differences in the total number of relapses were again between CD and UC patients (Model 1: OR = 0.392, CI: 0.223–0.690; Model 2: OR = 0.426, CI: 0.241–0.752) and between those with active versus inactive disease at baseline (Model 1: OR = 2.769, CI: 1.660–4.619; Model 2: OR = 3.501, CI: 2.118–5.786).

**Table 5:** Total number of relapses in the IBD group (n = 59) over the 12-month period

	**0**	**1**	**2**	**3**	**4**	**5**
**Frequency (%)**	22(37)	11(19)	7(12)	3(5)	5(8)	11(19)

**Table 6:** Interactions between psychological variables and a total number of relapses in the IBD group (n_CD _= 32 and n_UC _= 27)^1^

	**Adjusted OR**^1^	**95% CI**	**p**
**Disease status at baseline**	3.07	1.650–5.738	<0.0001
**Female vs. male**	1.19	0.677–2.117	0.535
**Age**	0.993	0.972–1.013	0.472
**Years since Diagnosis**	0.991	0.961–1.021	0.536
**CD vs. UC**	0.382	0.198–0.736	0.004
**HADS Anxiety**	0.967	0.841–1.111	0.632
**HADS Depression**	1.057	0.919–1.215	0.438
**SF12 Mental**	1.020	0.973–1.070	0.409
**SF12 Physical**	0.984	0.950–1.019	0.357
**SCL90 Somatisation**	1.012	0.972–1.055	0.554
**SCL90 Obsessive-compulsive**	0.993	0.942–1.047	0.800
**SCL90 Interpersonal sensitivity**	1.022	0.976–1.069	0.358
**SCL90 Depression**	1.003	0.928–1.085	0.931
**SCL90 Anxiety**	1.040	0.989–1.092	0.124
**SCL90 Hostility**	1.030	0.982–1.081	0.220
**SCL90 Phobic anxiety**	0.999	0.964–1.037	0.976
**SCL90 Paranoid ideation**	0.996	0.961–1.033	0.848
**SCL90 Psychoticism**	0.989	0.936–1.044	0.689
**SCL90 GSI**	0.999	0.909–1.098	0.983
**SCL90 PST**	0.962	0.832–1.111	0.596
**SCL90 PSDI**	0.979	0.936–1.025	0.375

^1 ^The analysis controlled for disease activity at baseline, sex, age, years since diagnosis and subtype of IBD (CD or UC)

## Discussion

There have been few studies to prospectively evaluate the relationship between depression and anxiety and patients' clinical outcomes. While studies conducted with other disease groups [36-38] have shown a link between psychological status and clinical outcomes, this investigation has not confirmed this observation for a cohort of patients with chronic gastrointestinal diseases.

Of note, this observational cohort study was only the fourth prospective, but the first controlled, investigation into the relationship between psychological status and clinical outcomes in patients with inflammatory bowel disease [25-27]. In contrast to previous observations [26,27], here we found that depression/anxiety at baseline was not associated with a total number of relapses after a period of time. Nevertheless, our results are consistent with other findings [25]. Other investigators [26], however, did not specify the timeline of their study and so accurate comparisons cannot be made. Interestingly, though, these researchers [26] have observed that psychological status adversely affected clinical outcomes only in patients with Crohn's disease, but not in those with ulcerative colitis.

The study by Mittermaier et al. [27], lasted for 18 months and thus, was six months longer than the present investigation. This might, in part, explain why the researchers found a positive relationship between depression at baseline and poorer clinical outcomes after 18 months. On the other hand, North et al. [25] observed their cohort of IBD patients for longer (two years), yet they did not observe a link between depression and worse clinical outcomes. The latter study, however, included only 32 participants. It is probable that larger and longer lasting studies could help understand these findings.

Sample size in the present study is comparable to that of Mittermaier et al. [27] (n = 59 vs. n = 60, respectively). As mentioned above their observations lasted 18 months, however, of more relevance, they reported a far greater baseline prevalence of depression – 28% as compared to our cohort's prevalence of only 12% [27]. This indicates significant differences between their cohort and ours, which are likely to have contributed significantly to our different findings. Interestingly, Mittermaier's patients were all in remission at baseline [27] and thus, should have had a low rate of psychological problems, while in the current study although only 61% of IBD patients were in remission at recruitment our cohort had a markedly lower rate of depression. To gather precisely comparable data further studies which recruit only IBD patients in remission would be required. However, allowing for this discrepancy in cohorts, when our analysis controlled for disease activity at baseline it did not appear to impact the results.

The largest limitation of the study by Mittermaier et al. [27] is the use of the Beck Depression Inventory (BDI), which is not as precise as the HADS in screening for depression and anxiety in IBD. In fact, five items in the BDI overlap with somatic symptoms of IBD: appetite, weight loss (two questions), general fatigue and worries about health. This may mean they over-diagnosed depression [27]. North et al. [25], on the other hand, avoided this flaw by removing these overlapping items from their version of the BDI. Thus, even though they had a smaller sample size, they applied greater rigour and had a greater length of observation [25]. Their data, adds weight to our findings, as they [25] also found no link between psychological status and clinical outcomes in patients with IBD.

As regards the HCV group, no similar studies have been performed and thus, no comparisons are possible. With respect to the IBS group, based on conflicting data from the available studies [21-23], it remains difficult to determine whether depression and/or anxiety predict worse clinical outcomes. The present study, in contrast to previous investigations, suggests that there is no relationship, either positive or negative, between both anxiety and depression and clinical outcomes after 12 months in patients with IBS. However, the present study did not achieve the estimated sample size in either the HCV or IBS group leaving us with insufficient power to be secure in determining whether such a relationship really exists. Thus findings of this study should be interpreted with caution, especially with respect to the IBS and HCV groups.

Moreover, the three groups were not homogenous at baseline and in future studies it may be better if of all patients were either in remission or in relapse at baseline. Due to the limitations of our study, and the continuing uncertainty from the published literature, longer and larger prospective studies are needed to better understand the relationship between psychological variables and relapse of somatic symptoms in these patients.

The largest limitation of this study may be too short a time frame. It is a difficult issue, as patients with all three studied conditions relapse less frequently than once a year. In fact, over 5 years, 25% of HCV patients will clear the virus [39] and the rest will still be carriers. Similarly in IBS, in a two-year period, 2–18% of patients have worse symptoms, 30–50% have unchanged symptoms and in the remainder symptoms either disappear or significantly improve [23]. In IBD, in a 2-year period approximately 30% of patients relapse [40]. This fact may explain why patients with inactive disease at baseline were at lower risk of active disease at 12 months. Thus, prospective investigations should probably last at least five years to accurately observe the relationship between psychological status and clinical outcomes in these particular patient groups.

Moreover, the definition of remission/relapse and a method of its measurement necessarily varied in these three diseases. In HCV for example, remission was defined as a clearance of the virus as measured by the blood test. In IBS, there are no precise disease activity indices available and thus, in this study subjective standardised questions on patients' perception of symptoms were chosen to evaluate the remission/relapse status. Finally, in both subtypes of IBD, disease activity indices are in the form of questionnaires which have been previously validated and widely used. One can, thus, argue that comparisons of these three disorders in terms of their disease activity are not fully justified due to these methodological differences in the measurement of the remission/relapse status. However, we would argue that these are the relevant outcomes to compare as these measures address the clinically relevant outcomes which both doctors and patients with these diseases seek.

Thus, despite its limitations, this study clearly showed that there is a need for more research exploring a link between psychological status and clinical outcomes. In light of the findings of the current study, longer lasting studies with larger and more homogenous groups of patients are clearly warranted.

## List of abbreviations

IBD: inflammatory bowel disease; CD: Crohn's disease; UC: ulcerative colitis; IBS: irritable bowel syndrome; HCV: hepatitis C; HADS: Hospital Anxiety and Depression Scale; HADSAnxiety: Hospital Anxiety and Depression Scale Anxiety subscale; HADSDepression: Hospital Anxiety and Depression Scale Depression subscale; SCL90: SCL-90-R Symptom Checklist; SCL90ANX: Anxiety; SCL90DEP: Depression; SCL90GSI: Global Severity Index; SCL90HOS: Hostility; SCL90IS: Interpersonal sensitivity; SCL90OC: Obsessive compulsive; SCL90PAR: Paranoid Ideation; SCL90PHOB: Phobic anxiety; SCL90PSDI: Positive Symptom Distress Index; SCL90PST: Positive Symptom Total; SCL90PSY: Psychoticism; SCL90SOM: Somatization; SF12: Short Form 12 Health Survey; MCS: Mental Component Summary; PCS: Physical Component Summary; CDAI: Crohn's Disease Activity Index; SCCAI: Simple Clinical Colitis Activity Index

## Competing interests

The study was funded by the Discipline of General Practice and the School of Psychology at the University of Adelaide, and the Department of Gastroenterology, Hepatology and General Medicine at the Royal Adelaide Hospital from International Postgraduate Research Scholarship. No industry sponsorship was involved. Authors are unaware of any conflicts of interest. However, Antonina Mikocka-Walus would like to acknowledge she received travel support from Ferring Pharmaceuticals. Professor Deborah Turnbull would like to acknowledge that she has received research support from AstraZeneca Pty Ltd, Aventis Pharma Pty Ltd, Bayer Australia Ltd, and Pfizer Pty Ltd. Dr Jane M. Andrews would like to acknowledge that she has been a consultant for Schering Plough and Pharmatel Fresenius Kabi. She has also received speaker's fees, travel and research support from AstraZeneca, Ferring, Orphan, Jannsen-Cilag, Schering Plough and Pharmatel Fresenius Kabi and Abbott. Professor Gerald Holtmann would like to acknowledge that he has been a consultant for Abbott, Altana, AstraZenecka, Takeda, Janssen-Cilag, Steigerwald, Knoll, and Novartis. He has also received research support from Altana, Ardeypharm, Deutsche Forschungsgemeinschaft, the NHMRC Australia, Zeria, and Novartis.

## Authors' contributions

The authors contributed equally to this work.

AMW contributed to the conception of this manuscript, designed it, collected data, performed analysis and interpreted data, and wrote the first and final drafts.

DT contributed to the conception of this manuscript, revised it critically and gave the final approval of the version to be published.

NM contributed to the study design, revised the manuscript critically and contributed to the final draft.

IW contributed to the study design, revised the manuscript critically and contributed to the final draft.

GH contributed to the study design, revised the manuscript critically and contributed to the final draft.

JA assisted with study design, recruitment, contributed to the conception of this manuscript, revised the manuscript critically and contributed to the final draft.

All authors read and approved the final manuscript.
